# Conformational Flexibility and Local Frustration in the Functional States of the SARS-CoV-2 Spike B.1.1.7 and B.1.351 Variants: Mutation-Induced Allosteric Modulation Mechanism of Functional Dynamics and Protein Stability

**DOI:** 10.3390/ijms23031646

**Published:** 2022-01-31

**Authors:** Gennady Verkhivker

**Affiliations:** 1 Keck Center for Science and Engineering, Graduate Program in Computational and Data Sciences, Schmid College of Science and Technology, Chapman University, Orange, CA 92866, USA; verkhivk@chapman.edu; Tel.: +17-14-516-4586; 2 Department of Biomedical and Pharmaceutical Sciences, Chapman University School of Pharmacy, Irvine, CA 92618, USA

**Keywords:** SARS-CoV-2 spike protein, ACE2 host receptor, mutational variants, conformational dynamics, local frustration, mutational scanning, protein stability, allosteric interactions

## Abstract

Structural and functional studies of the SARS-CoV-2 spike proteins have recently determined distinct functional states of the B.1.1.7 and B.1.351 spike variants, providing a molecular framework for understanding the mechanisms that link the effect of mutations with the enhanced virus infectivity and transmissibility. A detailed dynamic and energetic analysis of these variants was undertaken in the present work to quantify the effects of different mutations on functional conformational changes and stability of the SARS-CoV-2 spike protein. We employed the efficient and accurate coarse-grained (CG) simulations of multiple functional states of the D614G mutant, B.1.1.7 and B.1.351 spike variants to characterize conformational dynamics of the SARS-CoV-2 spike proteins and identify dynamic signatures of the functional regions that regulate transitions between the closed and open forms. By combining molecular simulations with full atomistic reconstruction of the trajectories and the ensemble-based mutational frustration analysis, we characterized how the intrinsic flexibility of specific spike regions can control functional conformational changes required for binding with the host-cell receptor. Using the residue-based mutational scanning of protein stability, we determined protein stability hotspots and identified potential energetic drivers favoring the receptor-accessible open spike states for the B.1.1.7 and B.1.351 spike variants. The results suggested that modulation of the energetic frustration at the inter-protomer interfaces can serve as a mechanism for allosteric couplings between mutational sites and the inter-protomer hinges of functional motions. The proposed mechanism of mutation-induced energetic frustration may result in greater adaptability and the emergence of multiple conformational states in the open form. This study suggested that SARS-CoV-2 B.1.1.7 and B.1.351 variants may leverage the intrinsic plasticity of functional regions in the spike protein for mutation-induced modulation of protein dynamics and allosteric regulation to control binding with the host cell receptor.

## 1. Introduction

The coronavirus SARS-CoV-2 infection is transmitted when the viral spike (S) glycoprotein binds to the angiotensin-converting enzyme 2 (ACE2) host receptor, leading to the entry of S protein into host cells and membrane fusion [1,2]. The full-length SARS-CoV-2 S protein consists of amino (N)-terminal S1 subunit and carboxyl (C)-terminal S2 subunit, where S1 is involved in the interactions with the host receptor and includes an N-terminal domain (NTD), the receptor-binding domain (RBD), and two structurally conserved subdomains CTD1 and CTD2 (Figure S1). Structural and biochemical studies established that the mechanism of virus infection may involve conformational transitions between distinct functional forms of the SARS-CoV-2 S protein in which the RBDs continuously switch between “down” and “up” positions [3,4,5,6,7,8,9,10]. The cryo-EM structure of the SARS-CoV-2 S trimer revealed a spectrum of closed states that included a structurally rigid closed form and more dynamic closed states preceding a transition to the fully open S conformation [5]. Protein engineering and structural studies also showed that specific disulfide bonds and proline mutations can modulate stability of the SARS-CoV-2 S trimer [6] and lead to the thermodynamic shifts between the closed and open forms [7,8,9]. Dynamic structural changes that accompany SARS-CoV-2 S binding with the ACE2 host receptor were described in cryo-EM experiments, showing a cascade of conformational transitions from a compact closed form, weakened after furin cleavage, to the partially open states, and, subsequently, the ACE2-bound open form thus priming the S protein for fusion [10]. The cryo-EM studies and biophysical studies of SARS-CoV-2 S trimer examined conformational flexibility and distribution of S trimers in situ on the virion surface, showing that spontaneous conformational changes and population shifts between different functional states are maintained in different biological environments and can coexist with receptor-induced structural adaptation, reflecting the intrinsic conformational landscapes of the SARS-CoV-2 S proteins [11,12].

The emergence of variants of concern (VOCs) with the enhanced transmissibility and infectivity profile, including D614G variant [13,14,15,16], B.1.1.7 (alpha) [17,18,19,20], B.1.351 (beta) [21,22], B.1.1.28/P.1 (gamma) [23] and B.1.1.427/B.1.429 (epsilon) variants [24,25] have attracted enormous attention in the scientific community and a considerable variety of the proposed mechanisms explaining functional observations from structural and biochemical perspectives. S-B.1.1.7 variant of the SARS-CoV-2 has 9 of the 17 mutations (Δ69-70 deletion, Δ144 deletion, N501Y, A570D, D614G, P681H, T716I, S982A, D1118H) in the S protein, featuring N501Y mutation, which can increase binding affinity with ACE2 while eliciting immune escape from RBD-targeting antibodies [17,18,19,20]. S-B.1.351 variant is characterized by 21 mutations with 9 mutations (L18F, D80A, D215G, R246I, K417N, E484K, N501Y, D614G, and A701V) in the spike protein, of which three mutations (K417N, E484K, and N501Y) are located in the RBD and increase the binding affinity for the ACE receptors and induce significant immune escape [21,22]. The cryo-EM studies provided atomistic characterization of the closed and open SARS-CoV-2 S trimers for these variants, suggesting subtle structural and functional effects of mutations that can modulate dynamics and stability of the closed and open forms, increase binding to the human receptor ACE2, and confer resistance to neutralizing antibodies [26,27,28,29]. The cryo-EM structures of the S-B.1.1.7 variant in the apo form identified four distinct conformational substates, all describing one or two RBDs in the open conformation without populating the fully closed state [30].

Importantly, these variants of the SARS-CoV-2 share D614G mutation, which is also individually linked with the enhanced infectivity, following the evidence of the mutation enrichment via epidemiological surveillance [13,14,15,16]. The initial structural studies showed that the D614G mutation can act by shifting the population of the SARS-CoV-2 S trimer from the closed form (53% of the equilibrium) in the native spike protein to a widely-open topology of the “up” protomers in the D614G mutant, with 36% of the population adopting a single open protomer, 39% with two open protomers and 20% with all three protomers in the open conformation [31]. These SARS-CoV-2 S ectodomain structures included two mutational regions in which the S1/S2 cleavage site residues 682-RRAR–685) are replaced by GSAS motif and two stabilizing prolines inserted at positions K986 and V987 to prevent conformational change to the post-fusion state [31]. The S-GSAS construct showed similar structural, antigenic, and stability behavior as the S-GSAS/PP construct, which included the K986P and V987P mutations. The cryo-EM structures of the S-GSAS/D614G mutant revealed the increased population of the 1-RBD-up open form as compared to the closed state in the S-GSAS/D614 structure [32]. The electron microscopy analysis confirmed the higher percentage (84%) of the 1-up RBD conformation in the S-G614 protein [33]. Functional studies showed that the S-G614 mutant exhibited a greater infectivity than the S-D614 protein, which was attributed to the greater stability of the S-G614 mutant and led to the reduced S1 subdomain shedding [34]. The increased stability of the D614G mutant was inferred from the recent cryo-EM structures of a full-length unmodified S-G614 trimer, which can reversibly adopt an all-down closed state and 1 RBD-up open conformation [35].

A significant heterogeneity and plasticity of the conformational landscapes for the SARS-CoV-2 S proteins was recently unveiled in the large-scale structural investigation that reported 38 cryo-EM structures of the B.1.351, B.1.1.28/P.1, B.1.617.2 (Delta), and B.1.617.1 (Kappa) variants, featuring a broad spectrum of the RBD-up conformations intrinsically present in the apo form [36]. In particular, S-B.1.351 variant displayed a highly populated fully open state with all three RBDs in the up conformation in addition to significant fractions of the partially open states (1 RBD-up or 2 RBD-up) [36]. Importantly, among all the examined variants only S-B.1.351 exhibited a fully open 3 RBD-up conformation that resembles the structure of the fully open S-G614 variant. It was suggested that the increased conformational diversity and a greater variability of the open states can allow SARS-CoV-2 S variants to adapt their responses and allow for escape immunity from common vaccines and different classes of monoclonal antibodies. Biophysical studies using microscale thermophoresis [37] and surface plasmon resonance (SPR) experiments [38] established that SARS-CoV-2 B.1.1.7 and B.1.351 spike variants bind human ACE2 with increased affinity. These studies suggested that the N501Y and S477N mutations enhance transmission primarily by enhancing binding, and that the K417N/T mutations facilitate immune escape, and the E484K mutation enhances binding and immune escape [38]. By engineering SARS-CoV-2 S proteins with different combinations of RBD variant mutations, cryo-EM structural analysis and biochemical assays systematically dissected the mutational effect on the ACE2 affinity and antibody evasion [39]. This study showed that N501Y, E484K, and L452R mutations can modulate, either directly or allosterically, the increased ACE2 binding affinity, while E484K, L452R, and K417N/T mutations tend to primarily compromise productive antibody binding and induce immune escape. Collectively, the rapidly growing body of structural and biochemical studies broadened our understanding of the conformational diversity and adaptability of the open states for the SARS-CoV-2 S variants, suggesting that coronaviruses have a broad potential to exploit synergistic effects of mutations to readily incur structural changes and modulate binding affinity with the ACE2 receptor and antibodies without a substantial loss of function [40]. The detection of common mutational changes such as D614G, E484K, N501Y and K417N that are shared among major circulating variants B.1.1.7, B.1.351, and B.1.1.28/P.1 indicated that these positions can be particularly critical for modulation of the SARS-CoV-2 S protein responses and may induce immunity escape from vaccines and different classes of monoclonal antibodies [41,42,43,44]. Together, these studies unveiled a complex balance among mutational variants in which individual modifications often act cooperatively to enhance or reduce protein stability, modulate binding to the ACE2 receptor, and confer immunity resistance to neutralizing antibodies [45].

Here, we employed computer simulations of the S-G614, S-B.1.1.7 and S-B.1.351 trimers in the closed and open states to systematically characterize the effect of mutational variants on conformational dynamics, and protein stability, as well as allosteric changes driven by collective motions and controlled by the regulatory hotspot centers. Computer simulations and protein modeling played an important role in shaping up our understanding of the dynamics and function of SARS-CoV-2 glycoproteins [46,47,48,49,50,51,52,53,54,55,56]. The latest extensive molecular dynamics (MD) simulations and free-energy landscape mapping studies of the SARS-CoV-2 S proteins and mutants detailed conformational changes and diversity of ensembles, further supporting the notion of enhanced functional and structural plasticity of S proteins [57,58,59,60,61,62,63]. Our recent studies offered evidence that the SARS-CoV-2 spike protein can function as an allosterically regulated machine that exploits plasticity of allosteric hotspots to fine-tune response to antibody binding [64,65,66,67,68,69]. These studies showed that examining allosteric behavior of the SARS-CoV-2 S proteins may be useful to uncover functional mechanisms and rationalize the growing body of diverse experimental data. In particular, using an atomistic-based model of signal transmission in the SARS-CoV-2 S proteins, we determined that the D614G mutation can exert its effect through allosterically induced changes on stability and communications in the residue interaction networks [70,71]. Previous computational studies also identified residues that exhibit long-distance couplings with the RBD opening that included sites harboring functional mutations D614G and A570D shared across SARS-CoV-2 S protein variants [59]. The free-energy landscapes of the S protein derived from MD simulations, together with nudged elastic pathway optimization mapping of the RBD opening, revealed a specific transient allosteric pocket at the hinge region, which is located near D614 position, influences RBD dynamics [60]. The energy analysis of the S-D614 and S-G614 proteins showed that local interactions near D614 position may be energetically frustrated and become stabilized in the S-G614 mutant through strengthening the inter-protomer association between S1 and S2 regions [63].

In the current study, we utilized molecular simulations of the studied SARS-CoV-2 S variants to perform the ensemble-based local frustration analysis of the S protein in different functional forms, and systematically characterize mutational adaptability of the spike residues. Conformational dynamics analysis and characterization of collective motions in the SARS-CoV-2 S variants are combined to identify the regulatory hotspots that control allosteric structural changes between the closed and opened states. We also perform a systematic mutational scanning of the SARS-CoV-2 S residues that are modified in the S protein variants in order to determine protein stability hotspots and identify potential energetic drivers underlying thermodynamic preferences of the B.1.1.7 and B.1.351 variants for the receptor-accessible open functional states. We show that modulation of the energetic frustration at the inter-protomer interfaces by mutational variants can serve as a mechanism for allosteric regulation in which mutations at the inter-protomer hinges of functional motions would modulate the inter-domain interactions, global changes in conformational mobility and the increased stabilization of the open form.

## 2. Results and Discussion

### 2.1. Atomistic Modeling and Simulations Reveal Distinct Conformational Flexibility Patterns of the SARS-CoV-2 S Mutant Variants

**Figure 1:**
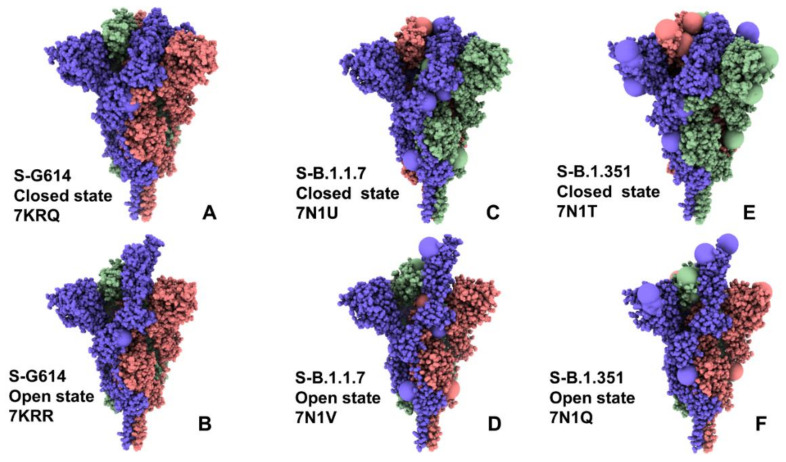
Cryo-EM structures of the SARS-CoV-2 S trimers used in this study. The S-G614 closed state (pdb id 7KRQ) (**A**) and S-G614 1 RBD-up open form (pdb id 7KRR) (**B**). The S-B.1.1.7 closed form (pdb id 7N1U) (**C**) and S-B.1.1.7 1 RBD-up open state (pdb id 7N1V) (**D**). The S-B.1.351 closed form (pdb id 7N1T) (**E**) and S-B.1.351 1 RBD-up open state (pdb id 7N1Q) (**F**). The structures are shown in full spheres and protomers A,B,C are colored in green, red and blue. The sites of mutational variants are shown in enlarged spheres and are colored based on the respective protomer. The mutational sites are D614G for S-G614 structures (panels **A**,**B**). The S-B.1.1.7 mutational sites are N501Y, A570D, D614G, P681H, T716I, S982A, and D1118H (panels **C**,**D**). The S-B.1.351 mutational sites are L18F, D80A, D215G, R246I, K417N, E484K, N501Y, D614G, and A701V (panels **E**,**F**). The rendering of SARS-CoV-2 S structures was performed using the interactive visualization program UCSF ChimeraX [72].

We employed multiple CG simulations to provide a comparative analysis of the conformational landscapes for the closed and open functional states of the S-G614 variant (Figure 1A,B), S-B.1.1.7 (Figure 1C,D), and S-B.1.351 (Figure 1E,F). While all-atom MD simulations with the explicit inclusion of the glycosylation shield could provide a rigorous assessment of conformational landscape of the SARS-CoV-2 S proteins, such direct simulations remain technically challenging due to the size of a complete SARS-CoV-2 S system embedded onto the membrane. We combined CG simulations with atomistic reconstruction and additional optimization by adding the glycosylated microenvironment. The closed and open forms of the S-B.1.1.7 and S-B.1.351 trimers are very similar to the S-G614 trimer structures (Figure 1A,C,E). The S1 subunit, which includes NTD (residues 14–306), RBD (residues 331-528), CTD1 (residues 528–591), and CTD2 (residues 592–685), showed a greater mobility level as compared to a more rigid S2 subunit (residues 686–1162). Common to these structures, 630-loop (residues 617 to 644) and FPPR segment (residues 828 to 853) are ordered in the RBD-down form, while the 630-loop becomes disordered in the 1-up protomer of the open state (Figure S1).

**Figure 2:**
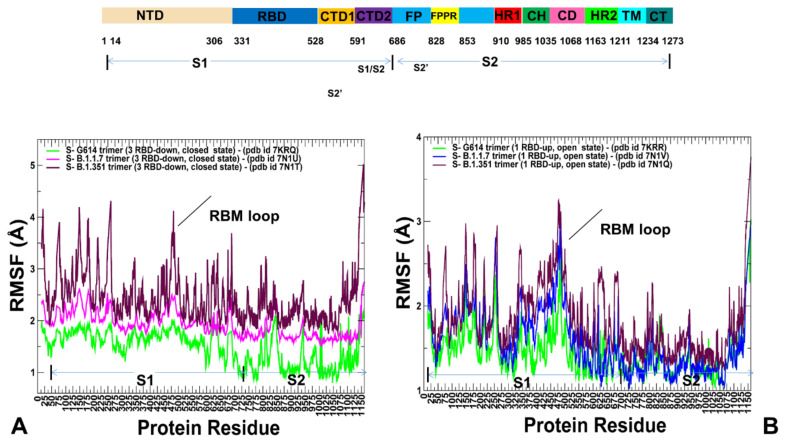
Conformational dynamics profiles obtained from simulations of the SARS-CoV-2 S protein variants. (**A**) The RMSF profiles obtained from simulations of the cryo-EM structures of SARS-CoV-2 S-G614 in the closed state, pdb id 7KRQ (green lines), S-B.1.1.7 closed form, pdb id 7N1U (blue lines), and S-B.1.351 closed state, pdb id 7N1T (maroon lines). (**B**) The RMSF profiles obtained from simulations of the cryo-EM structures of S-G614 in the open 1 RBD-up state, pdb id 7KRR (green lines), S-B.1.1.7 open 1 RBD-up form, pdb id 7N1V (blue lines), and S-B.1.351 open 1 RBD-up state, pdb id 7N1Q (maroon lines). The position of the RBM motif is indicated by an arrow. The S1 subunit (residues 14–685) and S2 subunit (residues 686–1163). The S1 domains include NTD (residues 14–306), RBD (residues 331–528), CTD1 (residues 528–591), CTD2 (residues 592–685). The S2 subunit contains upstream helix (UH), fusion peptide (FP), fusion peptide proximal region (FPPR), heptad repeat 1 (HR1), central helix region (CH), connector domain (CD), heptad repeat 2 (HR2), transmembrane domain (TM) and cytoplasmic tail (CT) regions. The S2 domains and functional regions of the simulated structures include UH (residues 736–781), FPPR segment (residues 828–853), HR1 (residues 910–985), CH (residues 986–1035), CD (residues 1035–1068), HR2 (residues 1069–1163).

Interestingly, the structural stability of the S2 subunit is especially apparent in the closed form of the S-G614 variant (Figure 2A). A comparative analysis of the dynamics profiles showed important differences in conformational flexibility of the S-G614, S-B.1.1.7 and S-B.1.351 variants in the closed states, despite very similar structural organization of the trimers (Figure 2A). In particular, the conformational fluctuations significantly increased in the S-B.1.351 closed state, suggesting that mutations of this variant could promote conformational flexibility and facilitate transitions to the partially open form (Figure 2A). Of particular interest are the large thermal fluctuations of the NTD regions and the receptor binding motif (RBM) (residues 424–494) where one of the key mutational sites E484 showed a considerable variability. In some contrast, the conformational mobility of these positions in the S-G614 and S-B.1.1.7 closed states may be more moderate. The enhanced mobility of this functional RBD region is important to enable spontaneous transitions between the closed and open forms required for engagement with the ACE2 receptor. It is worth noting that the conformational dynamics profiles also revealed the increased thermal “breezing” that is spread across both S1 and S2 subunits in the S-B.1.1.7 and S-B.1.351 closed states (Figure 2A), providing support for the notion that these mutational variants may perturb and destabilize the closed form, thereby shifting the equilibrium to a more dynamic open form [34,35,36]. The root mean square fluctuation (RMSF) profiles for the 1 RBD-up open states were similar for S-G614 and S-B.1.1.7 conformations, while larger fluctuations were observed in the S-B.1.351 structure (Figure 2B). These findings suggest a generally greater variability in the functional states of the S-B.1.351 trimer. It is important to stress that the conformational dynamics profiles describe the mean residue-based thermal fluctuations obtained by averaging over 1000 independent CG simulations. To highlight differences in mobility of the SARS-CoV-2 S variants, we also performed a simple statistical analysis, and computed averages and standard deviations of the RMSF values associated with different variants in the closed and open state. In the closed S conformations, the average RMSF values and standard deviation progressively increase from S-G614 to other variants and become considerably larger for S-B.1.351 (Table S1). The convergence of CG simulations was assessed by using the average Spearman’s correlation coefficient (*r_s_*) for residue mobility between different simulations based on the respective RMSF profiles [73] (Table S1). The reported average correlation coefficients for residue mobility between different CG simulations correspond to the mean values obtained from pairwise comparisons of 100 independent trajectories for each studied system. The average statistics showed a relatively high average Spearman’s correlation for S-G614 trajectories (*r_s_* = 0.835 for the closed state and *r_s_* = 0.804 for the open state) (Table S1). For more dynamic S-B.1.1.7 and S-B.1.351 variants, this correlation was somewhat lower but still statistically significant (*r_s_* = 0.745 for the S-B.1.1.7 open state and *r_s_* = 0.718 for the S-B.1.351 open state), owing to greater diversity and heterogeneity of open conformations (Table S1). The global similarity of conformations generated in CG simulations was obtained by computing the average root mean square deviation (RMSD) between the snapshots in the trajectories (Table S1). We measured the average similarity index of conformations using mean values of the pairwise RMSD computations for 100 independent trajectories in each studied system. The respective values reflected the diversity of open conformations, especially for the S-B.1.1.7 and S-B.1.351 variants.

**Figure 3:**
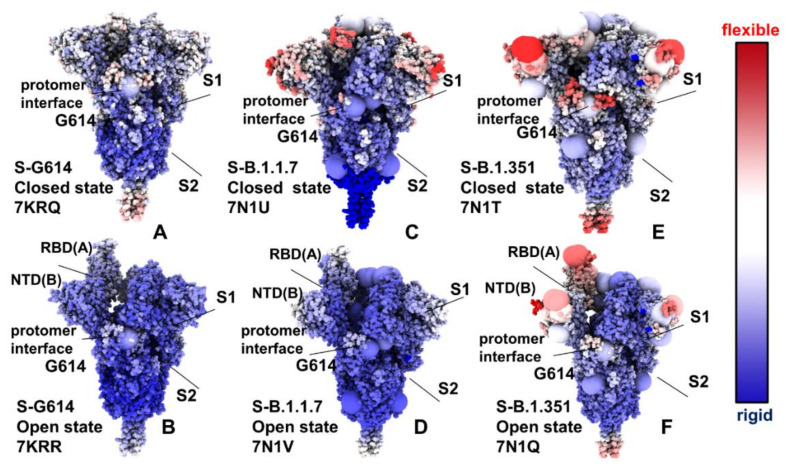
Structural maps of the conformational mobility RMSF profiles for the SARS-CoV-2 S protein variants. The RMSF profiles used for structural mapping are presented in Figure 2. The structural maps are projected onto the original cryo-EM structures. The conformational dynamics map for the S-G614 closed state (pdb id 7KRQ) (**A**) and S-G614 1 RBD-up open form (pdb id 7KRR) (**B**). Structural maps of the conformational mobility for the S-B.1.1.7 in the closed form (pdb id 7N1U) (**C**) and S-B.1.1.7 in the 1 RBD-up open state (pdb id 7N1V) (**D**). Structural maps of the conformational mobility for the S-B.1.351 in the closed form (pdb id 7N1T) (**E**) and S-B.1.351 in the 1 RBD-up open state (pdb id 7N1Q) (**F**). The sites of mutational variants are shown in enlarged spheres and are colored based on the respective protomer. The mutational sites are D614G for S-G614 structures (panels **A**,**B**). The S-B.1.1.7 mutational sites are N501Y, A570D, D614G, P681H, T716I, S982A, and D1118H (panels **C**,**D**). The S-B.1.351 mutational sites are L18F, D80A, D215G, R246I, K417N, E484K, N501Y, D614G, and A701V (panels E,F). The structures are in sphere-based representation and are rendered using UCSF ChimeraX [72] with the rigidity-to-flexibility sliding scale colored from blue (highly rigid) to red (highly flexible). The S1 and S2 subunits are indicated by arrows. The NTD(protomer B)-RBD(protomer A) interfaces and inter-protomer interfaces near G614 site are also indicated by arrows, and annotated.

Structural maps of the conformational dynamics profiles illustrated the increased mobility of the closed states for the S-G614, S-B.1.1.7 and S-B.1.351 variant trimers, while showing moderate flexibility of the open states (Figure 3). Of particular notice is a significant softening of the closed S-G614 and S-B.1.351 conformations. In these closed states, we detected the increased fluctuations in both S1 and S2 subunits (Figure 3A,E). For the S-B.1.1.7 closed trimer, the fluctuations of the S2 regions were smaller and a more significant flexibility was observed for the NTD regions (Figure 3C). These results suggest that the increased preferences of the S protein variants towards 1 RBD-up open conformation may be partly caused by the increased mobility and destabilization of the closed forms. One of the key observations of the conformational dynamics analysis is a steady stabilization trend in the open forms of the S protein variants (Figure 3B,D,F). In particular, we found that S-G614 and S-B.1.1.7 conformations displayed a broad stabilization in both S1 and S2 subunits but pointed to plasticity at the inter-protomer interfaces particularly near D614G site. Noticeably, the S2 subunit regions displayed only small fluctuations and are considerably more stable than more dynamic S1 domains (Figure 3B,D,F). The conformational variability in the NTD/RBD regions progressively increased in the open states from S-G614 (Figure 3B) to S-B.1.1.7 (Figure 3D) and S-B.1.351 variants (Figure 3F). Structural mapping of the conformational dynamics profiles also highlighted the increased flexibility at the S1/S2 interfaces and near inter-protomer boundaries, which may promote RBD movements in the open conformations for the S-B.1.1.7 and S-B.1.351 variants. The observed dynamic signatures of the S protein variants imply the greater diversity of the RBD-up conformations. Interestingly, the observed plasticity of the open conformations in the S-B.1.351 variants is consistent with the experimentally confirmed structural heterogeneity of the RBD-up conformations that was revealed in the recent cryo-EM studies, where the fully open 3 RBD-up state coexists with multiple substates of 1 RBD-up and 2 RBD-up conformations [36]. Accordingly, our findings agree with the experimental studies indicating that the greater variability of the open states can allow S-B.1.351 variant to readily adapt a spectrum of specific RBD-up conformations, thus providing a plausible mechanism for enhancing binding with the ACE2 receptor while modulating the immunity response.

By highlighting the sites of mutational variants, we gained some additional insight into mutation-induced modulation of conformational mobility in the S proteins. In particular, it is instructive to examine dynamic changes in the inter-protomer interface regions near G614 (Figure 3). This key mutational site shared by all studied variants experienced appreciable fluctuations in the 3 RBD-down states of the S-G614 (Figure 3A) and S-B.1.351 variant (Figure 3E), which may reflect the functional impact of mutations on perturbing the closed conformations and promoting transitions to an open form. The increased mobility of mutational sites is particularly apparent in the open state of the S-B.1.351 variant (Figure 3F), where all the modified positions L18F, D80A, D215G, R246I, K417N, E484K, N501Y, D614G, and A701V displayed a significant plasticity.

**Figure 4:**
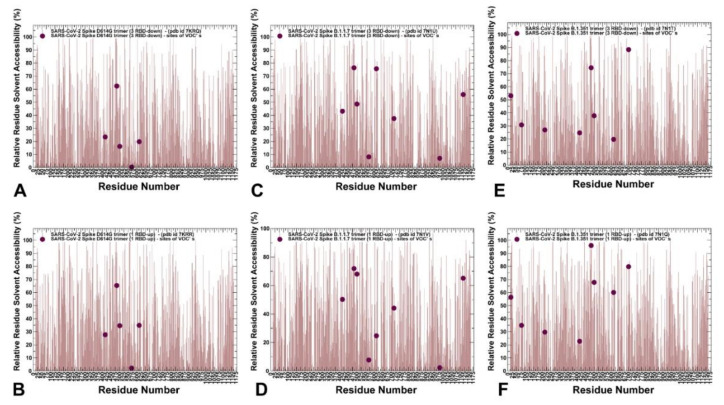
The relative residue-based solvent accessibility profiles for the SARS-CoV-2 S protein variants averaged over simulation trajectories. The absolute Accessible Surface Area (ASA) of each wild-type residue undergoing variation has been computed using the GetArea program [75]. The RSA values are obtained dividing absolute ASA values by residue-specific maximal accessibility values, as extracted from the Sander and Rost scale [76]. The residue-based RSA profiles for the S-G614 closed state (pdb id 7KRQ) (**A**) and 1 RBD-up open form (pdb id 7KRR) (**B**). The positions of K417, E484, N501, A570, and D614G are shown in maroon-colored spheres. The RSA profiles for SARS-CoV-2 S-B.1.1.7 closed form (pdb id 7N1U) (**C**) and 1 RBD-up open state (pdb id 7N1V) (**D**). The positions of K417, E484, N501Y, A570D, D614G, T716I, S982A, and D1118H are shown in maroon-colored spheres. The RSA profiles for SARS-CoV-2 S-B.1.351 closed form (pdb id 7N1T) (**E**) and 1 RBD-up open state (pdb id 7N1Q) (**F**). The positions of L18F, D80A, D215G, K417N, E484K, N501Y, D614G, and A701V are shown in maroon-colored spheres.

To characterize the ensemble-based structural environment of the mutational sites in the S proteins, we evaluated relative solvent accessibility (RSA) of protein residues. A residue-specific local RSA measure is defined as the ratio of the observed solvent-accessible surface area for a residue to the expected unfolded state value for that amino acid type [74,75]. The RSA values can be used as proxy for predicting intrinsic residue flexibility and the extent of conformational changes that may be induced by mutational variants. According to this model, residues are considered to be completely solvent-exposed if the ratio value exceeds 50%, to be buried if the ratio is less than 20%, and to be designated as partially buried for residues with 20% < RSA < 50%. We monitored the RSA values for the S protein residues averaged over simulation trajectories (Figure 4). For the S-G614 ensemble of closed conformations, only E484 residue is solvent-exposed, while K417 and N501 are buried (Figure 4A). The functional position A570 is totally buried and for the mutational variant position G614 the RSA value of ~19.8% is right at the border line between buried (RSA < 20 %) and partially buried sites (20% < RSA < 50%), reflecting some level of plasticity at the inter-protomer interface near the G614 l site (Figure 4A). In the S-G614 ensemble of open states, residues K417 (RSA = 27.7%) and N501 (RSA = 34.6%) become partially buried, while E484 (RSA = 65.4%) is solvent-exposed and the inter-protomer A570 position remains totally buried (RSA = 2.2%) (Figure 4B). Notably, the mutational site G614 becomes only partially buried (RSA = 35%) to the proposed scale, which reflected conformational flexibility of the RBD-up protomers and widening of the inter-protomer interfaces near the mutational site G614 (Figure 4B). Strikingly, in both closed and open states of the S-B.1.1.7 variant, A570D and S982A positions remain completely buried, featuring RSA < 10% (Figure 4C,D). At the same time, the functional RBD sites K417, E484, and N501Y become solvent-exposed, particularly E484 and N501Y positions with RSA > 70% (Figure 4C,D). Interestingly, while the G614 position is solvent-exposed in the closed state of S-B.1.1.7 variant, this site becomes partially buried in the open state, reflecting structural rearrangements of the inter-protomer interfaces near the G614 site. Notably, the degree of solvent exposure for the mutated RBD sites K417N, E484K, and N501Y appreciably increased in the S-B.1.351 states, particularly in the open form featuring RSA = 96% for E484K and RSA = 68% for N501Y positions (Figure 4E,F). The level of solvent accessibility is also markedly increased for the inter-protomer G614 site in the open state (RSA = 60%), which considerably increases its solvent exposure in the open state. These observations are consistent with the overall increase in the conformational flexibility in the S-B.1.1.7 and especially S-B.1.351 variants, as both closed and open forms of these variants displayed a considerable plasticity in the functional RBD regions.

### 2.2. Collective Modes of the SARS-CoV-2 S Protein Variants Reveal Role of A570D and D614G in the Hinge Regions Controlling Transitions between Open and Closed States

**Figure 5:**
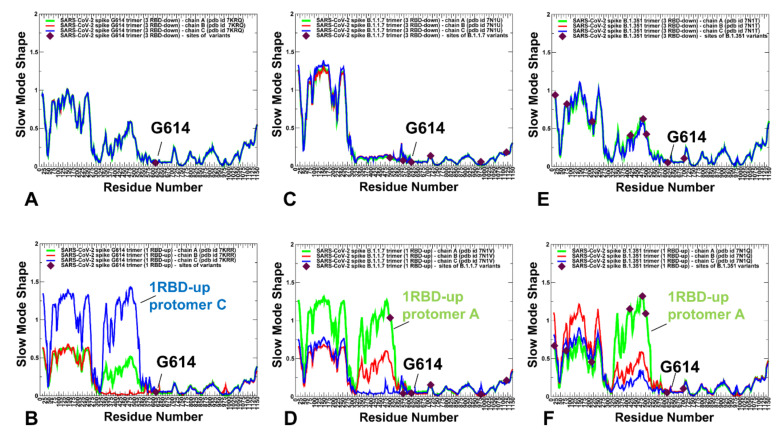
The slow mode mobility profiles of the SARS-CoV-2 S trimer structures. Based on the covariance matrix, by conducting PCA, the eigenvalues and the eigenvectors describing the collective protein dynamics modes are obtained. The slow mode shapes represent the displacements along slow mode eigenvectors and correspond to the cumulative contribution of the slowest three modes. The slow mode mobility profiles for the cryo-EM structures of the S-G614 in the closed state, pdb id 7KRQ; (**A**), S-G614 in the 1 RBD-up state, pdb id 7KRR (**B**), S-B.1.1.7 in the closed form, pdb id 7N1U (**C**), S-B.1.1.7 in the o1 RBD-up form, pdb id 7N1V (**D**), S-B.1.351in the closed state, pdb id 7N1T (**E**), and S-B.1.351 in the 1 RBD-up state, pdb id 7N1Q (**F**). The slow mode profiles for protomer chains A, B and C are shown in green, red and blue lines respectively. The 1 RBD-up protomer for the S-G614 open structure corresponds to protomer C (blue lines, panel **B**), the 1 RBD up protomer for the S-B.1.1.7 open structure corresponds to protomer A (green lines, panel **D**), and the 1 RBD up protomer for the S-B.1.351 open structure corresponds to protomer A (green lines, panel **F**). The position of D614G is shown in maroon-colored spheres for S-G614 on panels (**A**,**B**). The positions of K417, E484, N501Y, A570D, D614G, T716I, S982A, and D1118H are shown in maroon-colored spheres for S-B.1.1.7 on panels (**C**,**D**). The positions of L18F, D80A, D215G, K417N, E484K, N501Y, D614G, and A701V are shown in maroon-colored spheres for S-B.1.351 on panels (**E**,**F**).

To identify hinge sites and characterize collective motions in the S-G614, S-B.1.1.7 and S-B.1.351 variants, we performed principal component analysis (PCA) of atomistic reconstructed trajectories derived from CG simulations. The key functional signature of collective dynamics in the closed and open states are the large displacements for the NTD and RBD regions that enable functional movements of RBDs to their erected, receptor-accessible conformations (Figure 5). The immobilized hinge regions are aligned with the local minima along the slow mode mobility profiles, while the regions undergoing concerted movements typically correspond to the local maxima of the profiles (Figure 5). The hinge positions in the closed forms of the S protein variants are conserved and are aligned with residues F318, A570, F592, Q613, G614 and Y855 (Figure 5A,C,E). These sites are involved in coordination of the inter-domain movements between RBD and NTD as well as the relative motions of S1 and S2 regions. Notably, A570 and F592 hinge residues are situated near the inter-domain S1–S2 interfaces and could act as regulatory switch centers governing the population shifts between closed and open forms. Strikingly, the positions of mutational changes A570D, D614G, T716I, S982A in the S-B.1.1.7 states (Figure 5C,D) and positions D614G and A701V in the S-B.1.351 conformations (Figure 5E,F) are aligned with the local minima along slow mode displacement profiles and correspond to immobilized positions in slow motions. At the same time, E484K and N501Y sites are prominently featured among local maxima for the S-B.1.351 profiles (Figure 5E,F) and may experience functional changes due to movements of the RBDs. In the S-G614 (Figure 5B) and S-B.1.1.7 open states (Figure 5D), not only the RBD-up protomer undergoes functional motions, but significant displacements could occur for one of the RBD-down protomers. Moreover, for the S-B.1.351 open state (Figure 5F), functional movements were observed for all RBDs, even though the RBD-up displayed the larger magnitude of changes in the slow modes. This indicated that all S variants may promote movements of the RBD regions, which may increase in the S-B.1.1.7 and especially S-B.1.351 open conformations.

**Figure 6:**
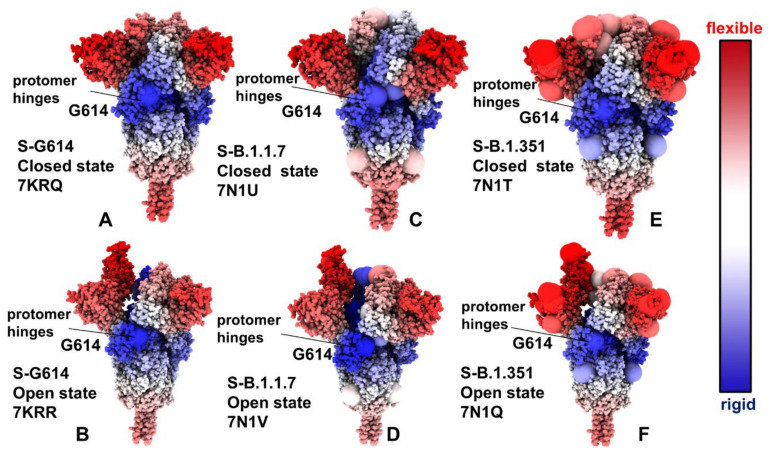
Structural maps of the slow mode mobility profiles for the SARS-CoV-2 S protein variants. The structural mapping of the slow mode mobility profiles projected onto the cryo-EM structure of S-G614 in the closed state (pdb id 7KRQ) (**A**) and the cryo-EM structure of S-G614 in the 1 RBD-up open state (pdb id 7KRR) (**B**). Structural mapping of the slow mode mobility profiles projected onto the cryo-EM structure of S-B.1.1.7 in the closed form (pdb id 7N1U) (**C**) and the cryo-EM structure of S-B.1.1.7 in the 1 RBD-up open state (pdb id 7N1V) (**D**). Structural maps of the slow mode mobility profiles are projected onto the cryo-EM structure of S-B.1.351 in the closed form (pdb id 7N1T) (**E**) and the cryo-EM structure of S-B.1.351 in the 1 RBD-up open state (pdb id 7N1Q) (**F**). The structural mapping is based on the computed cumulative contribution of the slowest three dynamics modes to the mobility of residues, as presented in Figure 5. The sites of mutational variants are shown in enlarged spheres and are colored based on the respective protomer. The mutational sites are D614G for S-G614 structures (panels **A**,**B**). The S-B.1.1.7 mutational sites are N501Y, A570D, P681H, T716I, S982A, and D1118H (panels **C**,**D**). The S-B.1.351 mutational sites are L18F, D80A, D215G, R246I, K417N, E484K, N501Y, D614G, and A701V (panels **E**,**F**). The structures are in sphere-based representation and are rendered using UCSF ChimeraX [72] with the rigidity-to-flexibility sliding scale colored blue (highly rigid) to red (highly flexible). The inter-protomer interfaces and hinge near G614 site are indicated by arrows, and annotated.

Structural projection of the slow mode profiles (Figure 6) illustrated the role of mutational sites in guiding collective protein dynamics. Importantly, the G614 position is consistently featured as the key immobilized hinge site in both closed and open states of the S protein variants. Another interesting observation is the emergence of A570D and D614G mutational sites as the key inter-protomer hinges that can orchestrate functional movements in the S-B.1.1.7 conformations (Figure 6C,D). At the same time, structural maps for the S-B.1.351 conformations (Figure 6E,F) highlighted the role of the NTD sites (L18F, D80A, D215G) and especially RBD sites (K417N, E484K, N501Y) that belong to the moving regions in slow motions. Combined, these analyses suggest that mutational sites in the S-B.1.1.7 and S-B.1.351 variants can play a significant role in both regulation and execution of collective movements that drive functional transitions between the closed and open states. These revelations may be particularly important given that the low-frequency soft modes are cooperative and robust as they are determined by the protein fold topology. Furthermore, allosterically-driven conformational changes typically evolve along slow modes of motions intrinsically accessible to folded structures [77,78]. As a result, allosteric responses in proteins can be efficiently triggered when mutations modulate protein propensities for energetically favorable movement along the slow modes. The observed correspondence between mutational sites and regulatory hinge points driving collective motions indicated that the virus may target these functional positions to modulate global dynamic response and promote allosteric transitions to the open state required for efficient binding with the host-cell receptor.

### 2.3. Local Frustration Analysis of the SARS-CoV-2 S Conformational States: Mutational Frustration Neutrality of Variant Sites and Differential Frustration of the Closed and Open States

**Figure 7:**
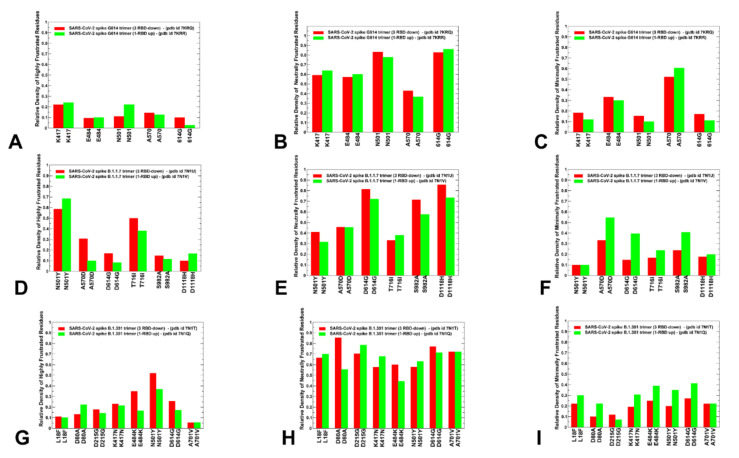
A comparison of the ensemble-averaged local mutational frustration between closed and open forms for sites of mutational variants and hinge positions of the S-G614 variant (**A**–**C**), S-B.1.1.7 variant (**D**–**F**) and S-B.1.351 variant (**G**–**I**). The relative densities of highly frustrated, neutrally frustrated and minimally frustrated residues are shown. The relative density index is shown in red bars for the closed states and in green bars for the open states.

We employed the conformational ensembles of the S-G614, S-B.1.1.7 and S-B.1.351 trimers to estimate the average local frustration profiles of the S residues and quantify the relationship between structural plasticity and mutational frustration. This analysis is based on scanning of the conformational ensembles by local frustratometer, which computes the local frustration index based on the contribution of a residue or residue pairs to the energy in a given conformation as compared to what it would contribute to decoy conformations [79,80,81,82,83]. The distribution of local mutational frustration showed a low relative density of highly frustrated population for residues targeted by mutations in the S trimer mutants (Figure 7). This is particularly apparent for the S-G614 conformations where K417, E484, N501, A570 and G614 sites featured a very small density of highly frustrated positions (Figure 7A). In some contrast, sites N501 and T716 in the S-B.1.1.7 conformations may be highly frustrated, while N501 and E484 may also feature an appreciable relative density of highly frustrated positions (Figure 7D). The role of frustration in the RBD regions is particularly interesting in light of recent evidence that the disordered or highly flexible regions can be critically important for mediating allostery and binding to multiple protein partners [84,85,86,87,88]. Notably, the population of highly frustrated conformations is relatively minor for all S conformations (Figure 7A,D,G). Importantly, we observed that sites of mutations in S-G614, S-B.1.1.7 and S-B.1.351 variants are largely associated with neutrally frustrated positions in both closed and open forms (Figure 7B,E,H).

A comparison of the local mutational frustration indexes for the functional positions, including sites of variants (K417, E484, N501) and hinge positions (A570 and G614), revealed interesting shifts between the closed and open forms of S-G614 trimer (Figure 7A–C). The high frustration density index for these positions is uniformly small, and similar in both closed and open conformations (Figure 7A). The relative density of neutral frustration is pronounced for all mutational sites, especially for the N501 and G614 positions, showing no appreciable differences between the closed and open states (Figure 7B). Notably, the hinge site A570 showed an appreciable density of minimal frustration, which is consistent with the regulatory role of this position in coordinating global allosteric changes. In general, sites of mutational variants located in the flexible RBD regions showed a propensity for neutral mutational frustration.

There are several revealing differences in the local frustration distributions of the S-B.1.1.7 (Figure 7D–F) and S-B.1.351 conformations (Figure 7G–I). Indeed, for the S-B.1.1.7 conformations, mutational positions D614G, S982A, D1118H are mostly neutrally frustrated (Figure 7E). At the same time, the key hinge position A570D showed a similar relative density of neutral and minimal frustration. Furthermore, the minimal frustration density distributions for A570D in the open state of the S-B.1.1.7 variant (Figure 7D–F) are also very similar to the corresponding frustration densities for A570 in the S-G614 open state (Figure 7A–C). According to our analysis, there is a greater degree of mutational and conformational plasticity near the D614G position that could allow for greater variability and diversity of the open states. Combined with the collective dynamics analysis, these observations suggest that moderately-to-minimally frustrated A570 and A570D hinge positions may allow for conformational switches at the inter-protomer interfaces that drive functional transitions. This is consistent with the experimental studies showing that A570D and D614G hinge positions in the S-B.1.1.7 protein form a molecular switch that incurs allosteric structural changes that can enhance the RBD motions [30]. In this S-B.1.1.7-specific switch, A570D forms the inter-protomer salt bridges with K854 and K964 that compensate for the loss of the salt bridge between D614 and K854 due to the D614G mutation [30].

In the S-B.1.351 conformations, we observed that sites targeted by mutations in the exposed RBD and NTD regions also featured neutral local frustration, suggesting a moderate mutational adaptability in these positions (Figure 7G–I). The relative density of minimal and high frustration for all mutational sites in the S-B.1.351 conformations is fairly low including NTD, RBD and S2 positions (Figure 7G,I). This could imply that the S-B.1.351 variant could promote a moderate level of conformational variability and functional adaptability in both closed and open states. The results offer an interesting rationale for the important role of A570D and D614G mutational sites in the S-B.1.1.7 and S-B.1.351 variants. These generally stable positions are involved in the inter-protomer hinge regions, that enable control functional transitions between closed and open forms. This minimal-to-neutral frustration level for A570D and D614G substitutions in the S-B.1.1.7 and S-B.1.351 conformations could allow for some mutational adaptability in the hinge position, which retains its regulatory role in the S variants, as confirmed by the structural and functional studies [30]. The generally prevailing pattern of frustration neutrality for sites targeted by mutations across all studied variants and significant contributions of high and neutral frustration density in the RBD sites E484 and N501 are important findings of this analysis, since local frustration is often an important mediator of allosteric changes. The relatively high frustration in these positions may allow for a discrete set of configurations involving local motions of the frustrated residues. Combined with modulation of the inter-protomer hinge regions by A570D and D614G positions, the local frustration in the RBD mutational sites could drive dynamical transitions between closed and open states, accompanied by local adjustments of the RBD residues.

The relationship between frustration in proteins and their function has been explored in illuminating studies by Wolynes [89]. The protein folding landscape theory established that while minimally frustrated interactions may have evolved in proteins to enable strongly funneled landscapes, a number of functional regions and specific protein sites could have been selected to be frustrated to allow for modulation of global motions and binding adaptability with various interaction partners. In this context, our results suggest that the detected neutral frustration patterns could allow for suboptimal inter-protomer interactions and adaptable substates to efficiently control access and binding affinity with the host-cell receptor ACE2.

### 2.4. Mutational Scanning of Protein Stability of the SARS-CoV-2 S-614 Conformational States Reveals Energetic Effects of the D614G Mutation

**Figure 8:**
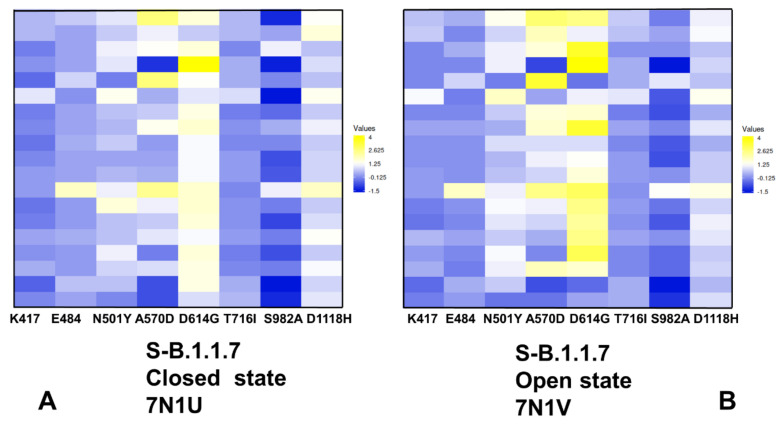
Ensemble-based mutational profiling of the SARS-CoV-2 S-B.1.1.7 protein stability. The mutational scanning heatmaps are shown for the closed state (**A**) and open state (**B**). The heatmaps show the computed binding free-energy changes for 19 single mutations on the sites of variants. The squares on the heatmap are colored using a 3-colored scale—from blue to white and to yellow, with yellow indicating the largest unfavorable effect on stability. The standard errors of the mean for binding free-energy changes were based on five independent CG trajectories for each of the S-B.1.1.7 states and different number of selected samples from a given trajectory (500, 1000 and 2000 samples) are ~0.11–0.24 kcal/mol using averages over different trajectories and ≤0.15 kcal/mol from computations based on different number of samples from a single trajectory.

**Figure 9:**
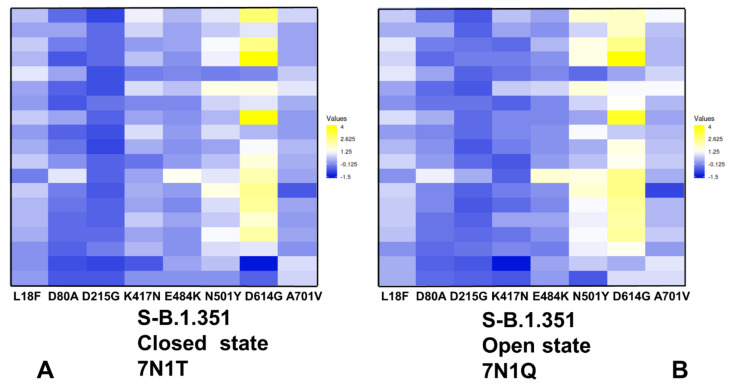
Ensemble-based mutational profiling of the SARS-CoV-2 S-B.1.351 protein stability. The mutational scanning heatmaps are shown for the closed state (**A**) and open state (**B**). The heatmaps show the computed binding free-energy changes for 19 single mutations on the sites of variants. The squares on the heatmap are colored using a 3-colored scale—from blue to white and to yellow, with yellow indicating the largest unfavorable effect on stability. The standard errors of the mean for binding free energy changes were based on five independent CG trajectories for each of the S-B.1.351 states and different number of selected samples from a given trajectory (500, 1000 and 2000 samples) are ~0.18–0.27 kcal/mol using averages over different trajectories and ≤0.17 kcal/mol from computations based on different number of samples from a single trajectory.

Using the equilibrium conformational ensembles, we performed mutational scanning of the spike protein residues and mutational sensitivity analysis of the S-B.1.1.7 (Figure 8) and S-B.1.351 mutant variants in the closed and open states (Figure 9). The protein stability ΔΔG changes were computed by averaging the results of computations over 1000 samples obtained from simulation trajectories. We first examined the pattern of free-energy changes for the S-B.1.1.7 closed and open states (Figure 8). Interestingly, mutational scanning of A570D and D614G produced relatively few destabilization changes in the open state (Figure 8B). Hence, structural stability constraints tend to protect the key positions that are responsible for modulation of the inter-protomer interactions. This is consistent with the experimental structural studies [26] showing that A570D and D614G favor the acquisition of the open S-B.1.1.7 state. In some contrast, mutations in the functional RBD positions (K417, E484) and modifications of mutational variant sites T716I, S982A and D1118H resulted in minor energy changes in both closed (Figure 8A) and open S-B.1.1.7 conformations (Figure 8B). These results are also consistent with the experiments [26] showing that N501Y, T716I and D1118H mutations induce only minimal local conformational changes without affecting stability of the S protein.

Interestingly, S982A substitution in the S-B.1.1.7 conformations abolished hydrogen bonding between central helices of the S2 domain and the CTD1 region [26]. As a result, mutations of this position in the S-B.1.1.7 states resulted in moderate and often stabilizing changes, indicating a considerable mutational tolerance and conformational plasticity in this region (Figure 8).

The mutational cartography analysis revealed that A570D and D614G mutational positions in the S-B.1.1.7 conformations are the most sensitive to modifications that result in more significant destabilizing changes (Figure 8). Importantly, the effect of mutations in these positions on the protein stability is greater in the open state (Figure 8B). The S-B.1.1.7 open state featured a salt bridge involving interactions of A570D with K854 of the other protomer [70]. In the closed S-B.1.1.7 states A570D can form the inter-protomer interactions with K964 and N856 that together comprise an important hinge cluster. Functional dynamics analysis of slow modes confirmed the role of A570D as a potential regulatory switch that controls RBD movements. Although modifications of A570D are generally destabilizing, the range of free-energy changes associated with this position suggested a moderate level of residual energetic frustration and suboptimal interactions (Figure 8). These findings are consistent with a series of structural studies showing a moderate degree of conformational heterogeneity in the interprotomer interactions formed by A570D in different substates of the S-B.1.1.7 protein [70,71]. Interestingly, substitutions in the D614G position are more destabilizing in both closed and open S-B.1.1.7 states (Figure 8), pointing to dynamic rearrangements near D614G position. Together, A570D and D614G sites are involved in the inter-protomer interactions in the S-B.1.1.7 states and contribute to the hinge clusters that modulate RBD motions. The results of mutational scanning are supportive of the local frustration analysis that displayed neutral-to-minimal frustration densities for A570D and D614G positions in the S-B.1.1.7 state. We suggest that neutral-to-minimal level of energetic frustration and moderate conformational plasticity in these regions and near the inter-domain interfaces could allow for emergence of mutational variants in sites responsible for allosteric modulation of conformational transitions between closed and open S states.

Mutational scanning maps for variant positions in the S-B.1.351 conformations showed similar and moderate stabilization changes for the NTD variant L18F, D80A, and D215G. It is evident that many substitutions in G215 position can be in fact energetically favorable for the protein stability (Figure 9). This pattern for the NTD mutational sites is shared in the closed and open states, suggesting that these variants could promote destabilization of the S-B.1.351 conformations and increase mobility and conformational variability of the NTDs.

Moderate free-energy changes are also seen for K417N and E484K sites in the closed and open states. Interestingly, there is a clear difference in the mutational map for the N501Y position (Figure 9). In agreement with the experimental studies, N501Y may become less frustrated in the open state of the S-B.1.351 variant (Figure 9B) and allow for more optimal interactions of the RBD-up protomer with the host receptor. In both states of S-B.1.351 variant, we observed appreciable destabilization changes induced by modifications in the D614G position (Figure 9). This is consistent with the increased density of minimal frustration for this position in the open S-B.1.351 state. Mutational site A701V is located in the surface-exposed region of S2 and caused minimal structural changes [26]. Consistent with these experimental observations, modifications in this position have a minor effect on the protein stability. Importantly, the results show a significant difference between S-B.1.1.7 and S-B.1.351 structures in the mutational profiles for K417/E484/N501 positions (Figure 8 and Figure 9). We found that these K417N, E484K and N501Y sites can afford greater mutational tolerance and conformational plasticity in the S-B.1.351 conformations. This suggests that the level of mutational and conformational plasticity can progressively increase from S-G614 to S-B.1.1.7 and S-B.1.351 variants.

The results also suggest an interesting interplay between mutation-induced protein stability and local frustration patterns. It is interesting to examine these relationships in the context of allosteric regulation models centered on the energetic frustration that could emerge at the inter-domain interfaces [79,80,81,82,83]. In these models, activation or repression functions may be encoded in the conformational ensemble and reveal through frustration-based allosteric regulation of the inter-domain interactions. The energetic scanning and local frustration analyses revealed the inherent mutational plasticity of the sites targeted by circulating variants. Mutational positions that are involved in hinge clusters are characterized by some degree of energetic frustration, thereby allowing for allosteric couplings and modulation of the RBD motions leading to the greater diversity of RBD-exposed conformations. These results suggested that S protein variants may uniquely exploit the intrinsic conformational and mutational plasticity of the S proteins, which is broadly distributed and is characteristic of not only RBD regions but also present in the inter-protomer and inter-domain regions. We argue that S-B.1.1.7 and S-B.1.351 variants may leverage this plasticity to adopt a mechanism of frustration-based allosteric modulation at the inter-protomer interfaces to differentially control binding with the host cell receptor ACE2 and interacting proteins.

## 3. Materials and Methods

### 3.1. Structure Preparation and Analysis

The following cryo-EM structures of the SARS-CoV-2 S protein variants were used in molecular simulations: the S-D614G variant in the 3RBD-down closed state (pdb id 7KRQ), S-D614G variant in the 1 RBD-up form (pdb id 7KRR), S-B.1.1.7 variant in the 3 RBD-down closed form (pdb id 7N1U), S-B.1.1.7 variant in the 1 RBD-up open state (pdb id 7N1V), S-B.1.351 variant in the 3 RBD-down closed form (pdb id 7N1T) and S-B.1.351 variant in the 1 RBD-up open state (pdb id 7N1Q). (Table 1, Figure 1).

All structures were obtained from the Protein Data Bank [90,91]. During structure preparation stage, protein residues in the crystal structures were inspected for missing residues and protons. Hydrogen atoms and missing residues were initially added and assigned according to the WHATIF program web interface [92,93]. The structures were further pre-processed through the Protein Preparation Wizard (Schrödinger, LLC, New York, NY, USA) and included the check of bond order, assignment and adjustment of ionization states, formation of disulphide bonds, removal of crystallographic water molecules and co-factors, capping of the termini, assignment of partial charges, and addition of possible missing atoms and side chains that were not assigned in the initial processing with the WHATIF program. The missing loops in the studied cryo-EM structures of the SARS-CoV-2 S protein were reconstructed and optimized using template-based loop prediction approaches ModLoop [94], ArchPRED server [95] and further confirmed by FALC (Fragment Assembly and Loop Closure) program [96]. The side chain rotamers were refined and optimized by SCWRL4 tool [97]. The conformational ensembles were also subjected to all-atom reconstruction using PULCHRA method [98] and CG2AA tool [99] to produce atomistic models of simulation trajectories. The protein structures were then optimized using atomic-level energy minimization with composite physics and knowledge-based force fields, as implemented in the 3Drefine method [100]. The atomistic structures from simulation trajectories were further elaborated by adding N-acetyl glycosamine (NAG) glycan residues, and optimized. The glycosylated microenvironment for atomistic models of the simulation trajectories was mimicked by using the structurally resolved glycan conformations for 22 most occupied N-glycans [101,102] in each as determined in the cryo-EM structures of the SARS-CoV-2 spike S trimer in the closed state (K986P/V987P,) (pdb id 6VXX) and open state (pdb id 6VYB), and the cryo-EM structure SARS-CoV-2 spike trimer (K986P/V987P) in the open state (pdb id 6VSB).

### 3.2. Coarse-Grained Simulations

CABS-flex approach was employed in CG simulations that efficiently combine a high-resolution interaction model and efficient search protocol shown to recapitulate all-atom MD simulation trajectories and dynamic profiles of large biomolecules on a long time-scale [103,104,105,106,107,108]. In this model, the amino acid residues are represented by Cα, Cβ, the center of mass of side chains and another pseudo atom placed in the center of the Cα-Cα pseudo-bond. The CABS-flex approach implemented as a Python 2.7 object-oriented standalone package [107] was employed in this study. Conformational sampling in the CABS-flex approach is conducted with the aid of Monte Carlo replica-exchange dynamics and involves local moves of individual amino acids in the protein structure, and global moves of small fragments [103,104,105]. CABS-flex protocol, in which long series of random local Monte Carlo changes are performed, can accurately recapitulate all-atom long-time dynamics [73]. The default settings were used, in which soft native-like restraints are imposed only on pairs of residues fulfilling the following conditions: the distance between their *C*_α_ atoms was smaller than 8 Å, and both residues belong to the same secondary structure elements. The CABS-flex default distance restraints moderately penalize the position of restrained residues if their distance differs from the distance in the original cryo-EM structure, becoming more than 1 Å. In these settings loop regions are fully unrestrained.

A total of 100 independent CG-CABS simulations were performed for each of the studied systems. In each simulation, the total number of cycles was set to 10,000 and the number of cycles between trajectory frames was 100. The convergence of CG-CABS simulations was ensured by performing multiple long-series Monte Carlo runs followed by statistical analysis of the RMSF profiles obtained in each of 100 long trajectories. In this analysis, the average Spearman’s correlation coefficients were computed between the RMSF profiles for individual trajectories [73]. The reported average correlation coefficients represent the mean values obtained from pairwise comparisons of 100 independent trajectories for each of the studied structures of the S-G614, S-B.1.1.7 and S-B.1.351 variants (Table S1). MODELLER-based reconstruction of simulation trajectories to all-atom representation provided by the CABS-flex package was employed to produce atomistic models of the equilibrium ensembles for studied systems [107]. Principal component analysis (PCA) of reconstructed atomistic trajectories was derived from CABS-CG simulations using the CARMA package [109].

### 3.3. Mutational Scanning and Sensitivity Analysis

A systematic mutational scanning and sensitivity analysis of the functional RBD residues (K417, E484, N501), S-B.1.1.7 mutational sites (N501Y, A570D, P681H, T716I, S982A, and D1118H) and S-B.1.351 mutational sites (L18F, D80A, D215G, K417N, E484K, N501Y, D614G, and A701V).

Each position was systematically mutated using all 19 substitutions and corresponding protein stability changes were computed. The BeAtMuSiC approach was employed, which is based on statistical potentials describing the pairwise inter-residue distances, backbone torsion angles and solvent accessibilities, and considers the effect of the mutation on the strength of the interactions at the interface and on the overall stability of the complex [110,111,112]. If a free-energy change occurs between a mutant and the wild-type (WT) proteins ΔΔG = ΔG (MT)-ΔG (WT) > 0, the mutation is destabilizing, while when ΔΔG < 0 the respective mutation is stabilizing. We computed the ensemble-averaged free energy changes using 1,000 equilibrium samples from simulation trajectories of the S-G614, S-B.1.1.7 and S-B.1.351 variants in both closed and open states.

## 4. Conclusions

In this work, we combined molecular simulations and collective dynamics analysis with the ensemble-based frustration analysis to characterize conformational plasticity and functional adaptability of the closed and open states for the S-G614, S-B.1.1.7 and S-B.1.351 variant. The conformational dynamics analysis revealed progressively increased mobility of the S-B.1.1.7 and S-B.1.351 variant in the open states. Collective dynamics of the S protein variants confirmed a critical regulatory role of the A570D and D614G positions acting as components of hinge clusters controlling the transitions between closed and open forms. This analysis suggests that in the S-B.1.1.7 and S-B.1.351 variants all RBDs can experience significant functional movements including the RBD-down conformations. We found that mutations in the S variants may promote movements of the RBD regions, which may increase in the S-B.1.1.7 and especially S-B.1.351 conformations. The local frustration analysis shows a prevailing pattern of frustration neutrality for sites targeted by mutations across all studied variant and dynamic contributions of high and neutral frustration in the RBD sites E484 and N501 positions. A strong preference of the mutational sites towards neutrally frustrated environment may allow for suboptimal inter-protomer interactions and regulatory control of SARS-CoV-2 S binding with the ACE2 host cell receptor. In agreement with structural studies, the ensemble-based energetic scanning of protein stability for mutated positions in the S protein variants revealed that level of conformational and mutational plasticity can progressively increase from S-G614 to the S-B.1.1.7 and S-B.1.351 variants. We found that K417N, E484K and N501Y sites are relatively tolerant to modifications in the S-B.1.351 closed and open conformations. The results also suggest an interplay between mutation-induced protein stability, local frustration and allosteric modulation of the S protein dynamics. By combining conformational dynamics and local frustration analysis with the ensemble-based mutational scanning, our analysis demonstrated that A570D and D614G mutational sites emerge as key inter-protomer hinges that control functional movements and allosteric conformational changes in the S-B.1.1.7 variant (Figure 6C,D). The results suggest that mutational variants target key residues of the spike protein that coordinate an allosteric cross-talk between rigid hinge sites (A570D, D614G) and more flexible RBD mutational sites (K417N, E484K, and N501Y). We argue that using a frustration-driven allosteric mechanism, mutational variants can impose regulatory control over functional movements and conformational adaptability of the RBD regions, thus modulating the accessibility and binding affinity with the ACE2 receptor. The proposed mechanism of mutation-induced energetic frustration may result in the greater adaptability of the open form for SARS-CoV-2 B.1.1.7 and B.1.351 variants.

## Figures and Tables

**Table 1:** Structures of SARS-CoV2 spike protein structures examined in this study.

PDB Code	Description	RBDs Position
7KRQ	SARS-CoV- 2 Spike Protein Trimer D614G variant—closed form	3 down
7KRR	SARS-CoV- 2 Spike Protein Trimer D614G variant—open form	1 up
7N1U	SARS-CoV- 2 Spike Protein Trimer B.1.1.7 variant—closed form	3 down
7N1V	SARS-CoV- 2 Spike Protein Trimer B.1.1.7 variant—open form	1 up
7N1T	SARS-CoV- 2 Spike Protein Trimer B.1.351 variant—closed form	3 down
7N1Q	SARS-CoV- 2 Spike Protein Trimer B.1.351 variant—open form	1 up

## Data Availability

Not applicable.

## Supplementary Materials

The following supporting information can be downloaded at: https://www.mdpi.com/article/10.3390/ijms23031646/s1.

## Abbreviations

SARS	Severe Acute Respiratory Syndrome
RBD	Receptor Binding Domain
ACE2	Angiotensin-Converting Enzyme 2 (ACE2)
NTD	N-terminal domain
RBD	receptor-binding domain
CTD1	C-terminal domain 1
CTD2	C-terminal domain 2
FP	fusion peptide
FPPR	fusion peptide proximal region
HR1	heptad repeat 1
CH	central helix region
CD	connector domain
HR2	heptad repeat 2
TM	transmembrane anchor
CT	cytoplasmic tail
