# Supplementary material for: Conformational Flexibility and Local Frustration in the Functional States of the SARS-CoV-2 Spike B.1.1.7 and B.1.351 Variants: Mutation-Induced Allosteric Modulation Mechanism of Functional Dynamics and Protein Stability
Source: Int J Mol Sci. 2022 Jan 31;23(3):1646. doi: 10.3390/ijms23031646 (PMC8836237; doi:10.3390/ijms23031646)
Supplement: Supplementary file 1 [file ijms-23-01646-s001.zip › ijms-1534641-supplementary.pdf]

Supplementary Materials

Conformational Flexibility and Local Frustration in the Functional States of the SARS-CoV-2 Spike B.1.1.7 and B.1.351 Variants : Mutation-Induced Allosteric Modulation Mechanism of Functional Dynamics and Protein Stability

Gennady Verkhivker[1,2*]

[1] Graduate Program in Computational and Data Sciences, Schmid College of Science and Technology, Chapman University, Orange, CA 92866, United States of America

[2] Department of Biomedical and Pharmaceutical Sciences, Chapman University School of Pharmacy, Irvine, CA 92618, United States of America

* Correspondence: verkhivk@chapman.edu; Tel.: +1-714-516-4586 (G.V)

Table S1. Statistical analysis of the multiple CG simulations for the SARS-CoV-2 S protein variants. *

PDB code	RBDs position	Mean RMSF	Standard Deviation (unbiased)	Average Spearman's Correlation Coefficient	Average Similarity between 100 independent CG trajectories
7KRQ	3 down	1.475	0.223	0.835	0.845
7KRR	1 up	1.442	0.287	0.804	0.823
7N1U	3 down	1.916	0.346	0.786	0.774
7N1V	1 up	1.872	0.370	0.745	0.748
7N1T	3 down	2.441	0.529	0.749	0.758
7N1Q	1 up	1.940	0.534	0.718	0.731

* The average RMSF values and standard deviations are obtained from 100 independent CG simulations for each system. The average Spearman's correlation coefficients for residue mobility between different CG simulations correspond to the mean values obtained from pairwise comparisons of 100 independent trajectories for each studied system. The global similarity of conformations generated in CG simulations was obtained by computing the average RMSDs between the snapshots in the trajectories.

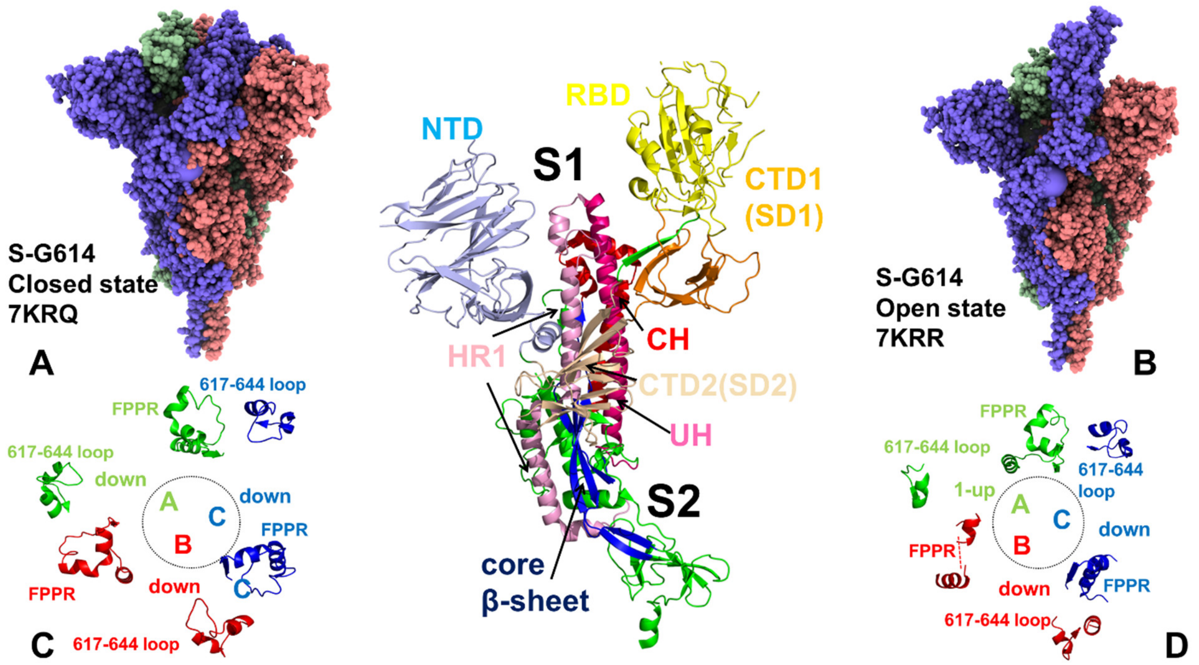

Figure S1. Structural annotation of the SARS-CoV-2 S protein domains and functional regions. The cryo-EM structures of the SARS-CoV-2 S-G614 closed state (pdb id 7KRQ) (**A**) and S-G614 1 RBD-up open form (pdb id 7KRR) (**B**). (Central panel) The domain organization for the full-length SARS-CoV-2 S protein. The highlighted S1 regions are NTD (14-306) in light blue; RBD (331-528) in yellow; CTD1 (528-591) in orange; CTD2 (592-686) in wheat color. The highlighted S2 regions are upstream helix (UH) (736-781) in red; HR1 (910-985) in pink; CH (986-1035) in hot pink; core β-sheet (711-736, 1045-1076) (in blue). Structural projection and annotation of the conformations for the 630 loop (residues 617-644) and FPPR (residues 828 to 862) mapped onto the SARS-CoV-2 S-G614 closed state (pdb id 7KRQ) (**C**) and the S-G614 1 RBD-up open form (pdb id 7KRR) (**D**).
